# Independent ischemic stroke risk factors in older Americans: a systematic review

**DOI:** 10.18632/aging.101987

**Published:** 2019-05-24

**Authors:** Jonathan Singer, Deborah Gustafson, Caroline Cummings, Aron Egelko, Jack Mlabasati, Alyssa Conigliaro, Steven R. Levine

**Affiliations:** 1 Department of Clinical Psychology, University of Nevada, Reno, NV 89557, USA; 2 Department of Neurology, Section for NeuroEpidemiology, State University of New York Downstate Medical Center, Brooklyn, NY 11203, USA; 3 State University of New York Downstate Medical Center, Department of Neurology and Stroke Center, New York City Health + Hospitals/Kings County, Brooklyn, NY 11203, USA; 4 Drexel University, Philadelphia, PA 19104, USA; 5 Department of Clinical Psychology, Hofstra University, Hempstead, NY 11549, USA

**Keywords:** stroke risk factors, ischemic stroke, cerebrovascular diseases, clinical studies, Framingham study, older adults

## Abstract

The Framingham Stroke Risk Profile (FSRP) is a validated model for predicting 10-year ischemic stroke risk in middle-aged adults, yet has not been demonstrated to consistently translate in older populations. This is a systematic review of independent risk factors measured among > 65 year olds, with subsequent first ischemic stroke, using PRISMA guidelines. We appraised peer-reviewed publications that included participants > 65 years old at risk assessment. Combined with other criteria, results were abstracted from 28 papers reporting six types of stroke risk factors: Serologic/Diagnostic, Conventional, Psychosocial, Genetic, Cognitive, and Antibiotic use. These studies demonstrated levels of serum androgens, C-reactive protein, and advanced glycation endproducts; thrombin generation; left ventricular mass; depressive symptoms; phosphodiesterase 4D single nucleotide polymorphisms; coagulation factor XII gene; peak thrombus generation; and lower cognitive functioning were independent risk factors for ischemic stroke in older adults. Plasma adipokines, free fatty acids and antibiotic use did not predict ischemic stroke. Purpose in life and APOEε2 allele were protective for ischemic stroke. This systematic review provides evidence of risk and protective factors for ischemic stroke in older cohorts that are not included in the FSRP. Further studies are needed to understand whether these factors are important enough to comprise a risk score.

## Introduction

Stroke is the 5th leading cause of death [1] and the leading cause of long-term disability in the United States [2]. The number of incident strokes is predicted to more than double between the years 2010 to 2050, with most strokes occurring in adults over the age of 75 years [3]. Additionally, this age group (>75 years) experiences more hospitalization stays and higher mortality [4] post-stroke. Despite these nuances of stroke risk and stroke outcomes at older ages, risk factors specific to this age group have not been well-studied. Instead, research has focused on examining risk factors in younger populations [5–8]. Therefore, accurate risk assessment tools and interventions to reduce stroke risk among older adults are lacking.

The most commonly used risk assessment tool for stroke prediction is the Framingham Stroke Risk Profile (FSRP) [5]. The FSRP was created using Cox proportional hazards regression modelling of Framingham Study data to identify factors that were most predictive of the 10-year probability of stroke. As a result, the FSRP includes age, systolic blood pressure, use of antihypertensive therapy, presence of diabetes mellitus, current cigarette smoking, history of cardiovascular disease (coronary heart disease, cardiac failure, or intermittent claudication), history of atrial fibrillation, and left ventricular hypertrophy by electrocardiogram [5]. Many studies have validated the FSRP for stroke prediction in elderly populations when FSRP components are measured in middle-age [6–8].

Given the increasing longevity of human populations with controlled multimorbidities [9], identifying potentially modifiable stroke risk factors in older age groups (> 65 years) will be vital to guide primary prevention and improve quality of care and quality of life to latest life, even with potential compression of morbidity. This systematic review appraises and then summarizes, the peer-reviewed published literature exploring independent risk factors, including components of the FSRP and others, in association with subsequent first ischemic stroke using the Preferred Reporting Items for Systematic Reviews and Meta-Analyses (PRISMA) guidelines. To the best of our knowledge, there are no other published systematic reviews exploring this topic.

## RESULTS

**Figure 1:**
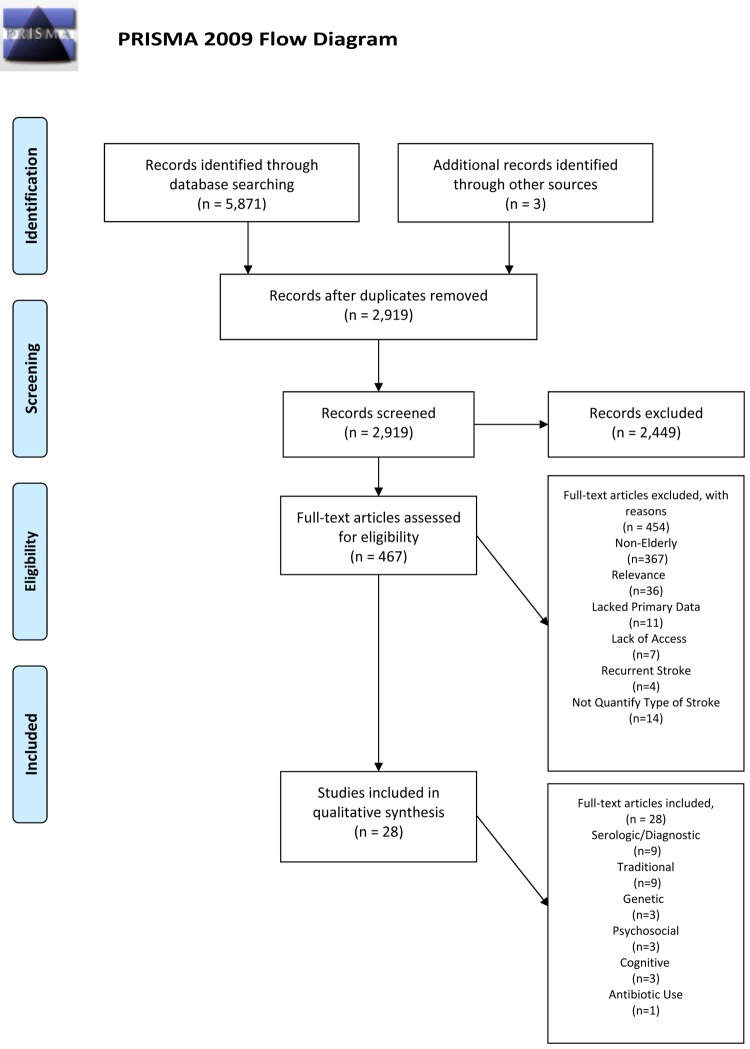
PRISMA 2009 Flow Diagram.

The initial search yielded 5,871 peer-reviewed publications, and 2,919 were reviewed after all duplicates were eliminated. All publication titles and abstracts were reviewed, which resulted in 467 full articles that fit criteria. Three additional articles were added based on citations from other papers. After reading the full articles, 425 articles were excluded based on our established exclusion criteria. Excluded were: 367 articles that did not focus on the elderly (<65 years), 47 that lacked primary data to address our study aim, 7 that were abstracts from conference presentations, 4 that focused on risk factors for recurrent stroke and 14 that did not ascertain type of stroke (i.e., ischemic; hemorrhagic). Thus, 28 papers published between 1992 and 2015, met our criteria and were included in our review (Figure 1, PRISMA). We extracted results from the 28 papers and categorized the articles into six areas: serologic/diagnostic risk factors (9 studies; see Table 1), conventional risk factors (9 studies; see Table 2), genetic risk factors (3 studies; see Table 3), psychosocial risk factors (3 studies; see Table 4), cognitive risk factors (3 studies; see Table 5), and antibiotics risk factors (1 study; see Table 5).

**Table 1:** Studies (n= 9) assessing serologic or diagnostic risk factors for ischemic stroke.

References	Aim of Study	Specific Risk Factor	Demographic	Outcomes
Kizer et al. (2014)	Examined relationship between Advanced Glycation Endproducts and incidence of IS.	CML	1,359 women; 752 men -Mean age 78.5	CML predisposes an individual for having an ischemic stroke in an older cohort
Cao et al., (2003)	Investigated CRP and IMT’s relationship to IS.	IMT CRP	3,142 women; 2,275 men Mean age 74.2	CRP’s is a risk factor when a person already has other IS risk factors
Sacco et al., (2004)	Investigated the relationship between tHcy and incidence of IS	tHcy	1,843 men; 1,086 men Mean age 69	tHcy had an independent association with adverse vascular outcomes, including IS; however, the mechanism underlying this relationship and the disparate outcomes by race has yet to be elucidated
Abbott et al., (2007)	Investigated relationship between endogenous hormones and risk of stroke	Serum Albumin; Sex Hormone Binding Globulin; Testosterone levels; Estradiol	Gender Breakdown NR Mean age 81.9	The critical deficit seems to be thermal and pinprick sensations Serum Albumin, Sex Hormone Binding Globulin, and Testosterone levels were not associated with risk of IS. Estradiol was associated with an increased IS risk
Shores et al. (2014)	Investigated relationship between endogenous hormones and risk of stroke	Testosterone; DHT	470 women; 562 men -Mean age 79	Low supraphysiologic levels of DHT are associated with increased risk of stroke
Khawaja et al., (2014)	Assessed Serum FFA levels association with IS	Serum FFA	2,594 women; 1,775 men -Mean age 75	FFA was not found to be associated with IS
Raipathak et al., (2011)	Assessed adipokines association with IS	Adiponectin, Leptin, and Resistin	Gender Breakdown NR -Mean 68.7	Resistin was the only adipokines found to predict risk for ischemic stroke
Saber et al., (2015)	Investigated the relationship between leptin levels and risk of IS	Leptin	470 women; 287 men -Mean age 79	No association between Leptin levels and IS.
Gardin et al., (2001)	Assessed the relationship between echocardiographic measures and risk of IS	M-Mode echocardiography	3,393 women; 2,495 men -Mean age 73	M-Mode echocardiography had only mild utility in assessing IS risk

CML: carboxymethyl-lysine; IS: ischemic stroke; CRP: C-reactive protein levels; IMT: carotid intima-media thickness; tHcy: serum total homocysteine; NR: not reported; DHT:dihydrotestosternone; FFA:free fatty acid

**Table 2:** Studies (n= 9) assessing conventional risk factors for ischemic stroke.

References	Aim of Study	Specific Risk Factor	Demographics	Outcomes
Mukamel et al. (2005)	Examined the association between alcohol use and ischemic stroke	Alcohol Use	Gender Breakdown NR -Mean age 72.4	The lowest risk of IS was found for consumers of 1-6 drinks per week (RR=.75; 95% CI=.53 to 1.06), in comparison to abstinent individuals (RR= .85; 95% CI=.63 to 1.13) and heavy drinkers (RR=1.03; 95% CI=.68 to 1.57). apoEε-4 gene modified the effect, as apoEε-4 carrier abstainers had a lower risk than apoEε-4 carrier drinkers, a pattern that was not found for apoEε-negative individuals.
Sacco et al. (1999a)	Assessed the association between alcohol intake and risk of ischemic stroke	Alcohol Use	384 women; 304 men -Mean age 70	The researchers concluded that alcohol intake may reduce the risk of suffering from IS when taken in moderation
Ottenbacher et al. (2004)	Examined diabetes as a risk factor for experiencing an IS	Diabetes	1,388 women; 969 men Mean age 72.6	Individuals with diabetes who were also taking insulin were more likely to suffer from an IS than their counterparts (HR=2.58; 95% CI=1.61 to 4.15), indicating that insulin use significantly increased diabetic individuals’ risk of suffering from an IS.
Naderi, Masoomi, Mozaffar and Malik (2014)	Investigated the relationship between AMI and IS	AMI	12,983 women;15,396 men -Mean age 74.27	AMI was significantly associated with an increased risk of suffering from an IS, especially in those individuals 65 years and older (AOR=1.62; 95% CI=1.56 to 1.88)
Lichtman, Krumbolz, Wang, Radford and Brass (2002)	Investigated the relationship between AMI and IS	AMI	54,400 women; 56,723 men Mean age 76	The risk of stroke after AMI was found to be substantial with 1 in 40 suffering an ischemic stroke within 6 months of their AMI
Abbott et al. (2001)	Examined the association between ankle/brachial blood pressure index (ABI) and risk of stroke, with 3-year and 6-year follow-up time points	ABI	2,767 men (no women) - Over 65, but mean age NR (Groups has mean age over 70 years old)	Individuals with low ABI had almost a 200% increase in risk of suffering from an IS (AHR=1.9; 95% CI=1.0 to 3.7), with the risk increasing with age. -Those in the oldest age group (85-93 years old) with low ABI had the highest risk of experiencing an IS
Seshardi et al., (2001)	Assessed the association between prior and current blood pressure levels on risk of IS	ASBP; DBP; PP	2,666 (70+) women; 1,586 men Over 70, but mean age NR	In the 70+ age group, ASBP, DBP, and PP were predictive of IS in women, however, only ASBP and DBP were predictive of IS in men
Colantonio, Kasl & Ostfield (1992)	Investigated PF’s relationship to IS	PF	1,659 women; 1,152 men Mean age 74	Low physical activity, a risk factor already identified for younger older adults (<75) for IS, is a risk factor for ischemic stroke in this older population (>75)
Sacco et al. (1998)	Investigated leisure time PF and IS	Leisure Time PF	597 women; 450 men Mean age 69.9	More leisure-time physical activities endorsed resulted in lower risk of IS in this older population

IS: ischemic stroke; AMI: acute myocardial infarction; ABI: ankle/brachial blood pressure index; NR: not reported; ASBP: antecedent systolic blood pressure; DBP: diastolic blood pressure; PP: pulse pressure; PF: physical functioning

**Table 3:** Studies (n= 3) assessing genetic risk factors for ischemic stroke.

References	Aim of Study	Specific Risk Factor	Demographics	Outcomes
Ferrucci et al. (1997)	Assessed risk of IS in individuals who were genotyped for the Apoε allele	ApoEε 2, 3ε and 4ε	1088 women; 576 men - Mean age 78.9	The presence of ApoEε2 allele was associated with a lower incidence of stroke in individuals 71-79 years old. No protective effect was seen in those over 80 years old nor was an association found between ApoEε3 or ApoEε4 and ischemic stroke with any age group.
Brophy et al. (2006)	Assessed Phosphodiesterase 4D SNPs association with IS	Phosphodiesterase 4D SNPs (9, 42, 175, 219, and 220)	All women -Mean age 73.9	Stratifying by hypertensive status yielded significant associations with IS for 4 polymorphisms in women without hypertension: SNPs 9, 42, 219, and 220 -Individuals who were hypertensive and had the SNP 175 gene showed higher rates of IS -Individuals who were not hypertensive and had the AC008828-2 gene had significantly higher rates of IS Individuals who were hypertensive and had the AC008828-2 gene did not predict higher risk of IS
Olsen et al. (2015)	Evaluated the relationship between SNPs in the Coagulation Factor XII gene (*F12)*, pTG, and IS risk	Coagulation Factor XII gene (*F12)*, pTG	Gender Breakdown NR -Over 65, but mean age NR (Groups has mean age over 70 years old)	pTG was found to be significantly associated with IS risk, and 2 *F12* SNPs were found to be associated with pTG. However, these SNPs were not independently associated with an increased incidence of IS

SNP: single nucleotide polymorphisms; IS: ischemic stroke; pTG: peak TG; NP: not reported

**Table 4:** Studies (n= 3) assessing psychosocial risk factors for ischemic stroke.

References	Aim of Study	Specific Risk Factor	Demographics	Outcomes
Arbelaez, Ariyo, Crum, Fried and Ford (2007)	Investigated the association between depression and IS	Depression	3,213 women; 2,312 men -Mean age 72.7	Older adults with clinical depression at baseline had a 32% increased hazard ratio for ischemic stroke (HR= 1.32, 95% CI=1.09 to 1.59)
Henderson et al., (2013)	Investigated psychosocial stress and IS mortality	Psychosocial stress; Depression	2,545 women; 2,133 men -Mean age 77.1	The findings suggest that depression alone is a risk factor, but, when accompanied by other proposed risk factors, its effect is diminished
Yu et al., (2015)	Investigated if lower purpose in life would predict increased risk of cerebral infarct	Purpose in life	310 women; 142 men -Mean age 83.0	Findings suggest that having a greater sense of purpose in life is associated with ≈50% reduced likelihood of cerebral infarcts

IS: ischemic stroke

**Table 5:** Studies (n= 4) assessing cognition/antibiotic use risk factors for ischemic stroke.

References	Aim of Study	Specific Risk Factor	Demographics	Outcomes
Ostir et al., (2003)	Investigated cognitive functioning of older Mexican Americans as a risk factor for IS	Cognitive Functioning	1,569 women; 1,113 men Mean age NR	Reported that cognitive functioning significantly predicted IS no matter what age group of the participant. For every 1 point increase in MMSE there was a 5% reduction in risk of ischemic stroke
Elkins et al., (2004)	Evaluated the relationship between cognitive functioning and IS	Cognitive Functioning	Gender Breakdown NR -Mean age 74.2 (high cognitive functioning group=73.4 yrs.; lower cognitive functioning group=75.0 yrs.)	Higher levels of cognitive functioning were associated with lower risk of IS A secondary analyses divided the individuals at baseline into quartiles based on the amount of stroke risk factors they endorsed and still found that no matter the amount of risk factors, higher cognitive functioning was associated with lower risk of IS
Ferruci et al., (1999)	Investigated the relationship between cognitive functioning and IS	Cognitive Functioning	3,323 women; 1,701 men -Mean age 78.5	Stroke incidence was lowest in those with normal cognitive functioning (12.1 per 1000 person), intermediate with moderate impairment (16.3 per 1000 person), and higher in those with severe impairment (30.9 per 1000 persons). The authors found the same results when controlling for age and gender.
Luchsinger et al., (2001)*	Assessed if short-term antibiotic use targeted at C. pneumoniae, a bacteria associated with chlamydia, decreases IS risk	Short-term antibiotic use targeted at C. pneumoniae	48,379 women; 86,789 men -Mean age 74	Antibiotic use was generally not found to be associated with stroke risk, as only 2 of the 6 antibiotics prescribed influenced the risk ratio: quinolones (HR=1.30; 95% CI=1.21 to 1.40) and cephalosporins (HR=1.17; 95% CI=1.10 to 1.25). The researchers concluded that antibiotic use does not reduce individuals’ risk of IS

IS: ischemic stroke; NR: not reported; MMSE: mini mental status exam

*Miscellaneous, not cognitive section

### Serologic or diagnostic risk factors

In recent years, investigating serologic biomarkers has positively impacted our understanding of the pathophysiology of ischemic stroke. We identified nine studies that investigated either serological or diagnostic risk factors for incident ischemic stroke (Table 1). The results were mixed. One study [11] assessed the relationship between advanced glycation endproducts (specifically carboxymethyl-lysine [CML]), and incident ischemic stroke. An adjusted cubic spline analysis was consistent with a linear relationship between CML and ischemic stroke. A multivariable hazard ratio (mHR) of 1.14 (95% CI, 1.01-1.28; p= 0.027) per 0.99 pmol/l of serum CML was reported. The authors concluded that CML is a marker of the vascular effects of advanced glycation end products, thus predisposing an individual to stroke.

Using a similar study design, associations between an established biomarker of inflammation, serum C-reactive protein levels (CRP) and carotid intima-media thickness (CIMT), a marker of atherosclerosis, were associated with incidence of ischemic stroke. Associations were examined after stratification by quartile of serum CRP levels [12]. Using the first quartile of CRP as the referent, the mHR for ischemic stroke by second, third, and fourth quartiles were 1.19 (95% CI, 0.92-1.53), 1.05 (95% CI, 0.81-1.37), and 1.60 (95% CI, 1.23-2.08), respectively. While CRP levels in the fourth, or or highest, quartile were an independent risk factor for ischemic stroke, the magnitude of the risk was attenuated when CIMT was added as a covariate to the model. The authors concluded that CRP is a risk factor for ischemic stroke when controlling for atherosclerosis markers, such as CIMT.

Serum total homocysteine (tHcy) [13], a known cardiovascular risk factor, was investigated in relation to ischemic stroke. A tHcy ≥ 15 umol/L was a significant and independent predictor of ischemic stroke risk, with a mHR of 2.01 (95% CI, 1.00-4.05).

Given interest in the role of estrogens and androgens in cardiovascular and cerebrovascular disease, two studies [14,15] investigated endogenous blood sex hormone or related protein levels and risk of ischemic stroke, yielding similar results. One study [14] investigated men of Japanese ancestry who were living in the United States, observing that serum levels of albumin, sex hormone binding globulin, and testosterone were not associated with risk of ischemic stroke. However, estradiol was associated with an over 2-fold increased risk for ischemic stroke, after adjusting for serum testosterone, hypertension, and diabetes (mHR 2.2, 95% CI, 1.5-3.4; p<0.001). The second study [15] investigated serum levels of testosterone and dihydrotestosterone (DHT) in association with ischemic stroke in men. Testosterone levels were again not associated with ischemic stroke risk. However, DHT levels that were either higher or lower than the reference value (50-75 ng/dL) were associated with ischemic stroke. Specifically, low (<25 ng/dL) DHT levels were associated with a mHR of 2.20 (95% CI, 1.18-4.12) and high (>100 ng/dL) DHT levels were associated with mHR of 1.89 (95% CI, 0.55-6.54). This overall U-shaped association of DHT with ischemic stroke risk, adjusting for covariates (e.g., age; gender; ever smoked), was significant (*p*=0.006).

As obesity is a known risk factor for stroke, adipose tissue-related metabolites and hormones were examined in relation to ischemic stroke in three studies [16–18]. One study [16] assessed serum free fatty acid (FFA) levels and found no association between FFA levels and incidence of ischemic stroke. A second case-control study [17] enrolled post-menopausal women who had experienced an ischemic stroke and assessed the association with three plasma-based adipokines: adiponectin, leptin, and resistin. After adjusting for body mass index (BMI) and other ischemic stroke risk factors, resistin was the only adipokine found to predict the odds of ischemic stroke (mOR, 1.61; 95% CI, 1.22-2.13; *p*<0.001). The third study [18] of both men and women investigated the relationship between leptin levels and risk of ischemic stroke and found no association. It should be noted that sex hormone metabolism changes in women and men as they age. Testosterone levels decline in men, however, most notably, in postmenopausal women, with cessation of ovarian function, the primary site of estrogen synthesis is adipose tissue [19–21]. Androstenedione is aromatized to estrone, which while having low activity in the presence of estradiol, becomes primary when estradiol production is diminished [19–21].

There was one study that evaluated a non-serological biomarker [22]. This study investigated subjects who had received baseline M-Mode echocardiography to assess the relationship between echocardiographic measures and risk of ischemic stroke. After adjusting for conventional risk factors, left ventricular (LV) mass was associated with an increased HR for ischemic stroke (mHR=3.36, 95% CI, 1.96-5.74). However, no specific pattern of LV hypertrophy conferred higher risk for ischemic stroke. Additionally, left atrial dimensions and the presence of mitral annulus calcification were not associated with ischemic stroke risk.

### Conventional

Risk factors (Table 2) for ischemic stroke that are deemed ‘conventional’, as identified in the Framingham Study [5], were evaluated. Nine studies were identified that measured these conventional risk factors in adults >65 years. These risk factors included alcohol intake [23,24], type 2 diabetes [25], myocardial infarction [26,27], blood pressure/pulse pressure [28,29], and physical functioning [30,31].

#### Alcohol use

One study examined the association between alcohol intake and ischemic stroke in a sample of Medicare-eligible older adults aged 65 years and older [23]. After an average of 9.2 years follow-up, alcohol intake was associated with a U-shaped risk for incident ischemic stroke. Specifically, the lowest risk of ischemic stroke was found for consumers of 1-6 drinks per week (mRR=0.75; 95% CI, 0.53-1.06), in comparison to abstinent individuals (mRR= 0.85; 95% CI, 0.63-1.13) and heavy drinkers (>14 drinks per week; RR=1.03; 95% CI, 0.68-1.57). Further, the APOEε4 gene allele modified this association. Among APOEε4 allele carriers, abstainers had a lower ischemic stroke risk compared to all categories of drinkers. This pattern was not observed for those who did not carry an APOEε4 allele. No other variables measured (e.g., systolic blood pressure, baseline hypertension, baseline atrial fibrillation, or blood lipids) influenced these relationships.

Another study [24] examined the association between alcohol intake and risk of ischemic stroke [24] in a multi-ethnic urban sample of stroke patients matched with community controls by sex, race/ethnicity, and within 5 years of age. In this sample, a J-shaped pattern was again found between alcohol intake and risk of ischemic stroke. However, the investigators used very different drinking categories compared to the aforementioned study. Moderate drinkers”, those who had at least one drink in the previous year but not more than 2 drinks per day, had the lowest risk of suffering from ischemic stroke. Moderate drinking was associated with a 50% reduction in stroke risk (adjusted odds ratio, AOR= 0.51, 95% CI, 0.39- 0.67). Heavy drinking (7 or more drinks per day) was reported in the paper to increase risk (AOR=1.63; 95% CI, 0.74-3.62) of stroke, albeit not significantly. Similar to the earlier study [23], type of beverage did not influence this result, nor did multivariate adjustment for hypertension, diabetes, cardiac disease, smoking, or BMI alter this association. Reformed heavy drinkers (lifetime average daily intake of > 5 drinks but current daily intake < 2 drinks) did not have a higher risk of stroke (OR= 0.66; 95% CI, 0.31-1.41).

#### Type-2 Diabetes

The only study meeting our eligibility criteria [25] that examined diabetes as a risk factor for ischemic stroke was conducted in a sample of Mexican-American individuals. Of 524 diabetic individuals, 12% (N=64) experienced a stroke within 7-months follow-up, in comparison to 7% (N=142) of those without diabetes. Furthermore, compared to those without diabetes, both non-insulin dependent (mHR=1.78; 95% CI, 1.32-2.42) and insulin –dependent (mHR=2.58; 95% CI, 1.61-4.15) diabetics were more likely to have an ischemic stroke.

#### Myocardial infarction

Cardiovascular events may precede and increase risk for cerebrovascular events. One study [26] aimed to understand the occurrence and predictive risk factors associated with experiencing a cerebrovascular event defined as ischemic stroke, hemorrhagic stroke and transient ischemic attack, after being hospitalized with acute myocardial infarction (AMI). AMI was associated with an increased risk of an ischemic stroke, especially among those aged 65 years and older (mOR=1.62; 95% CI, 1.56-1.88).

Another study [27] examined hospital data to assess the rate of an ischemic stroke admission within 6-months following an AMI-related hospitalization. These investigators also created a risk stratification score that identified individuals at greatest risk for 1) stroke after AMI and 2) recurrent stroke. Two and a half percent of AMI patients experienced an ischemic stroke within 6 months of hospital discharge. Age greater than 75 years (RR=1.29; 95% CI, 1.18-1.40), and presence of at least one comorbid condition (e.g., previous stroke (RR=1.75; 95% CI, 1.59-1.92), atrial fibrillation (RR=1.52; 95% CI, 1.39-1.67), history of peripheral vascular disease (RR=1.31; 95% CI, 1.18-1.47), diabetes (RR=1.26; 95% CI, 1.16-1.37), hypertension (RR=1.13; 95% CI, 1.04-1.23), and any frailty (RR=1.27; 95% CI, 1.16-1.38), increased AMI patients’ risk of ischemic stroke. Aspirin use at discharge (RR=0.86; 95% CI, 0.79-0.93) lowered risk by approximately 15%. Moreover, the risk of ischemic stroke post-AMI was substantial, affecting 1 in 40 within 6 months. Evaluation of a risk stratification score, albeit not described, allowed the researchers to conclude that previously confirmed risk factors for ischemic stroke, such as hypertension, diabetes, and peripheral vascular disease, extended to their sample of patients with a history of AMI.

#### Blood pressure/pulse pressure

Hypertension is a well-known risk factor for stroke [28,29]. The association of baseline blood pressure levels on 20-year risk of ischemic stroke was assessed in a sample of older adults [28]. Ischemic stroke occurred in 491 participants, of whom at baseline, 142 were age 60-69 years, 231 between 70-79 years, and 118 between 80-89 years. The risk of ischemic stroke associated with current and former high blood pressure decreased with age, with the highest systolic blood pressure-associated risk occurring at age 60-69 years and declining thereafter. Further, among those age 70-79 years, baseline systolic blood pressure, diastolic blood pressure, and pulse pressure were predictive of stroke in women, however, only systolic and diastolic blood pressure were predictive of stroke in men.

The association between ankle-brachial index (ABI) and risk of ischemic stroke, with 3-year and 6-year follow-up time points, has been reported [29]. ABI is a ratio of blood pressure taken from the ankle to the blood pressure in the upper arm. In a sample of 2,767 men between 71-93 years of age and living in Hawaii, 65 (2.3%) experienced a thromboembolic stroke (a subtype of ischemic stroke) by 6-year follow up. Compared to those with higher ABI, individuals with low ABI had an almost 2-fold increase in risk of ischemic stroke (Adjusted HR =1.9; 95% CI, 1.0-3.7), and risk increased with age. Specifically, those in the oldest age group (85-93 years) with low ABI, had the highest risk of ischemic stroke; and lower ABI was associated with earlier onset of stroke by over 6 months.

#### Physical functioning

Diminishing physical functioning has been shown to be a precursor to stroke. The Yale Health and Aging Project enrolled participants over the age of 65 years who were living independently (not living in a senior facility or institutionalized) and without history of stroke or transient ischemic attack (TIA) [30]. Participants were primarily female (59%) and Caucasian (79%) and followed for 6 years after baseline measurements. Physical functioning was measured using two different scales – the Katz Scale of Daily Living and the Roscow Scale. In separate analytic models, the two physical functioning scales showed that higher levels of physical functioning lowered 6-year risk of ischemic stroke after adjustment for sex and baseline age, diabetes, hypertension, and angina (mRR=1.49; 95% CI, 1.31-1.69).

The Northern Manhattan Stroke Study (NOMAS), an ongoing, prospective, population-based study, designed to determine stroke incidence, risk factors, and prognosis in an underrepresented population [31], examined whether leisure-time physical activity was associated with ischemic stroke. The authors measured leisure-time physical activity using an adapted questionnaire from the National Health Interview Survey of the National Center for Health Statistics, which queried participants about their engagement in 14 recreational activities (e.g., walking; golf) in the previous 2 weeks and, if so, the frequency at which they engaged. A higher frequency of self-reported leisure-time physical activities was associated with lower risk of ischemic stroke.

### Genetics

Table 3 describes results from studies that investigated presence of the APOEε2, ε3, and ε4 alleles in association with risk of ischemic stroke [32]. The presence of APOEε2 allele was associated with a lower incidence of stroke in individuals 71-79 years old. No protective effect was seen in those over 80 years old, nor was an association found between APOEε3 or APOEε4 and ischemic stroke in any age group. The APOEε2 allele has been shown to be protective for later-life outcomes such as Alzheimer’s disease and related dementias as well, which are also associated with vascular risk factors, including stroke. As aforementioned, APOEε4 status was an effect modifier in the analyses associating alcohol intake with stroke risk.

Two studies [33,34] analyzed single nucleotide polymorphisms (SNPs) in the phosphodiesterase 4D and AC008828-2 genes and risk of ischemic stroke. Both the phosphodiesterase 4D and AC008828-2 genes have been studied extensively with research showing their positive association with ischemic stroke risk [35]. Among women [33], 4 phosphodiesterase 4D SNPs were associated with both higher and lower ischemic stroke risk, after stratification by hypertension status. Notably, among women without hypertension, the SNPs rs9 (HR=0.48; 95% CI, 0.26-0.91), rs42 (HR=1.73; 95% CI, 1.10-2.70), rs219 (HR=1.73; 95% CI, 1.13-2.64), and rs220 (HR=1.56; 95% CI, 1.05-2.32) of the phosphodiesterase 4D gene were associated with ischemic stroke. The second study [34] evaluated the relationship between SNPs in the coagulation factor XII gene (F12*)*, peak thrombin generation (pTG) level, and ischemic stroke risk. pTG was associated with higher ischemic stroke risk (HR=1.09; 95% CI, 1.01-1.17; *p*=.03) and 2 F12 SNPs were associated with greater pTG. However, these latter SNPs were not independently associated with an increased incidence of ischemic stroke.

### Psychosocial

#### Depression

Depression has been reported as both cause and consequence of cardio- and cerebrovascular events [36]. A mediating role of inflammation has been hypothesized. The relationship between depressive symptoms and ischemic stroke after 11 years of follow-up in a prospective cohort study of adults aged 65 years and older at baseline (Table 4), showed a positive relationship between baseline depressive symptoms, measured by a modified 30-point Center for Epidemiologic Depression Scale (CES-D), and risk of ischemic stroke. Older adults with a clinically relevant depressive symptom burden (CES-D >8) at baseline had a 32% higher risk of ischemic stroke (crude HR=1.32; 95% CI, 1.09-1.59; mHR=1.26; 95% CI, 1.03-1.54).

Psychosocial distress, including depressive symptoms, in relation to incident ischemic stroke in older adults aged 65 and older has also been addressed in a 6-year longitudinal, population-based study [37]. Participants were recruited from three neighborhoods in Chicago. Depressive symptoms were measured via the CES-D. Results indicated that 408 individuals (11%) experienced an ischemic stroke. Baseline depressive symptoms increased risk for stroke 12% (HR 1.12; 95% CI, 1.01-1.24; p=0.03) after adjustment for race, age, and sex. However, with adjustment for additional stroke risk factors, including education, BMI, systolic blood pressure, smoking status, physical activity, chronic conditions (cardiovascular diseases; hip fracture; diabetes mellitus; cancer) and lipid-lowering medications, the association disappeared (HR=1.02; 95% CI, 0.091-1.15).

#### Psychological well-being

Not only are risk factors evaluated in relation to stroke, but protective factors as well. An ongoing prospective cohort study, the Rush Memory and Aging Project (MAP) [38], a sample of community-dwelling older adults, examined purpose in life (i.e., the sense that life has meaning and direction) as protective for cerebral infarctions. Participants were enrolled and followed until death, after which an autopsy was completed among those who consented. The study found that 25.3% of older adult participants had a clinical stroke, and that nearly twice as many had macroscopic or microscopic infarcts at autopsy (n=216; 47.7%). Purpose in life was measured by Ryff’s Purpose in Life Scale, a subscale of the subjective well-being scale. Greater purpose in life was associated with lower odds of macroscopic infarct (mOR=0.535; 95% CI, 0.346-0.826), with control for BMI, history of smoking, diabetes mellitus, and blood pressure. However, no association was found between purpose in life and microscopic infarcts (mOR=0.780; 95% CI, 0.495-1.229). A greater purpose in life was associated with fewer subcortical macroscopic infarcts (OR=1.13; 95% CI, 0.61-2.08), but not with cortical macroscopic infarcts (OR=0.51; 95% CI, 0.32-0.82). When further categorizing macroscopic infarcts into lacunar and non-lacunar infarcts, greater purpose in life was associated with fewer lacunar infarcts (OR=0.50; 95% CI, 0.30-0.84).

### Cognition

While it is well-known that stroke contributes to vascular cognitive impairments and disorders, it is less clear whether cognitive function predicts stroke. In a study [39] measuring cognition using the Mini Mental Status Exam (MMSE), a measure of global cognitive function, those scoring > 21 (range 0-30), denoting better cognitive function, were half as likely to have an ischemic stroke at seven year follow-up (HR=0.49; 95% CI, 0.35-0.69) compared to those scoring < 20. All participants had no history of stroke at baseline and were followed for seven years, with follow-up visits at 2, 5 and 7 years. Using MMSE has a continuous score, global cognitive function predicted ischemic stroke, irrespective of baseline age group (65-74; 75-84; >85 years). For every 1-point increase in MMSE there was a 5% reduction in ischemic stroke risk across all age groups (Table 5). While crude, the MMSE has been used in numerous epidemiological studies of elderly and has high construct validity [40],

In a similar study of participants > 65 years old [41], the relationship between cognitive function and incident ischemic stroke was evaluated. Another test of global cognitive function, the modified Mini Mental State Examination (3MS), was used. 3MS score was dichotomized to define those with high cognitive function (scores > 96 of 100) and low cognitive function (scores <96 of 100). Those with high cognitive function experienced a lower risk of ischemic stroke (OR=0.68; 95% CI, 0.52-0.88; *p*=.005). A secondary analysis was conducted after stratification by baseline quartile of stroke risk factor burden defined via systolic blood pressure, blood glucose level, presence of Type-2 diabetes, walking the previous day, and history of heart disease.

The association between cognitive function and ischemic stroke was evaluated in older Americans, >71 years [42]. The majority of the sample was female (66%) and White (95%), with a sub-sample of African Americans (5%). Seventy-nine percent of the sample scored in the normal range (7-9 correct answers) on the Pfeiffer’s Short Portable Mental Status Questionnaire (SPMSQ), 18% showed moderate impairment (4-6 correct answers) and 3% showed severe impairment (0-3 correct answers). Stroke incidence was lowest among those with normal cognitive functioning (12.1 per 1,000 persons), intermediate with moderate impairment (16.3 per 1,000 person), and highest in those with severe impairment (30.9 per 1,000 persons). Results were similar with control for age and sex.

### Antibiotic use as a risk factor

Infectious diseases, both bacterial and viral, are associated with higher cardiovascular and cerebrovascular disease risk. One study meeting our criteria [43] tested the hypothesis that short-term antibiotic use targeted at *Chlamydia pneumoniae*, decreases ischemic stroke risk. *C. pneumoniae* is associated with lung infections via airborne routes of transmission. Using a healthcare database sample of individuals over the age of 65 with an insurance claim for ischemic stroke, individuals with and without a pharmacy claim for an antibiotic prescription were compared. In a study of almost 200,000 claims, incident stroke was experienced by 7335 individuals over a mean follow-up period of 1855 ± 554 days (median=1159). In univariate analyses, general antibiotic use was not found to be associated with stroke risk. However, 2 of the 6 antibiotics prescribed were associated with a 20-30% higher stroke risk (cephalosporins (HR=1.17; 95% CI, 1.10-1.25) and quinolones (HR=1.30; 95% CI, 1.21-1.40). After multivariate adjustment for stroke risk factors such as diabetes, hypertension, hyperlipidemia, coronary artery disease, chronic obstructive pulmonary disease, and atrial fibrillation, only quinolones remained associated with stroke risk (HR=1.17; 95% CI, 1.09-1.26). The researchers concluded that antibiotic use does not reduce risk of stroke, although a higher risk may be evident among those using specific antibiotics. Given the potential chronic nature of *C. pneumoniae* infection, intermittent use of quinolones over time may be problematic.

## DISCUSSION

Several types of risk factors for ischemic stroke were evaluated in this review. A brief discussion of each type follows.

### Serologic or diagnostic risk factors

Serologic biomarkers have greatly expanded our understanding of the pathophysiology of ischemic stroke [44]. Reliable serologic diagnostic markers to predict ischemic stroke risk are of great interest given the relative ease by which they can be obtained. The studies included in this review found that several serologic markers, including CML, CRP, tHcy, estradiol, DHT and resistin, are associated with an increased risk of ischemic stroke. However, other serologic risk factors for ischemic stroke demonstrated in studies involving younger populations (<65 years) were not found to be significant, including serum albumin, SHBG, testosterone, leptin, and adiponectin. The imaging biomarker, M-Mode echocardiography, was also not associated [45]. While these serologic biomarkers may be informative for clinicians assessing ischemic stroke risk, the risk associated with each of these markers was relatively low. The physiologic basis underlying these associations requires further exploration, especially within older age groups, to identify more precise risk biomarkers.

### Conventional

Equivocal results for conventional risk factors among older adults were associated with ischemic stroke. While the studies reviewed provide insight, there are limitations to be considered. Up-to-date studies are needed, as many of the studies [23,24,27–31] reviewed were published between 1992 and 2005. Trends in both risk factors and outcomes due to, for example, secular trends, changes in diagnostic criteria, and diagnostic advances may influence observed associations. Moreover, as common in epidemiologic studies, subjective and/or retrospective measures of exposures may be biased. While still commonly used, some self-reported conventional risk factors are being tracked using new technologies (e.g., Ecological Momentary Assessment). This may help to advance exposure assessments. For example, both studies reviewed that examined alcohol use [23,24] were based on participants’ self-report of prior alcohol consumption that occurred more than a year before the interview (i.e., “how much alcohol did you drink two years ago?”). Another study [24] relied on participants’ self-report of diabetes and ischemic stroke and did not corroborate the responses with medical records. Furthermore, one of these studies [i.e., 31] did not report a risk estimate, making it difficult to evaluate and compare results. Notwithstanding, these published reports significantly add to our knowledge of stroke risk, and if they are replicated in accordance with our criteria, will provide more confidence in the observed association.

### Genetic

Four studies were identified that assessed genetic predisposition as a risk factor for ischemic stroke based on candidate genes and previously published SNPs. Studies continue to examine the role of other SNPs and genetic markers with ischemic stroke. The four studies identified here found that four phosphodiesterase 4D SNPs were associated with ischemic stroke after stratification by hypertensive status, pTG was associated with increased risk of ischemic stroke and an association between two F12 SNPs were associated with pTG. The APOEε2 allele was associated with a lower incidence of stroke in individuals 71-79 years old, but was not associated with stroke among individuals over the age of 80 years old.

### Psychosocial

The extant literature regarding psychosocial risk factors for stroke in older adults was reviewed and suggested multiple risk and protective factors. Our search results led to publications on depressive symptoms and purpose in life. Overall the findings suggest that the presence of depressive symptoms in older adults are associated with stroke incidence, though comorbid risk factors reduced this association; and that purpose in life may be protective against future stroke.

### Cognition

There are clear weaknesses in the studies examining cognition in association with future ischemic stroke in older populations. For example, the scales used, such as the MMSE and 3MS, have weak psychometric properties and small sample sizes comprise the published studies reviewed. However, taken together, some studies provide evidence that higher global cognition is associated with lower risk for incident ischemic stroke. This points to the potential importance of cognitive and brain reserve in relation to both cognition and cerebrovascular events.

## Conclusions and Limitations

There are several limitations of this systematic review. First, this review only focuses on articles that were published in English. Despite this language restriction, more than four thousand articles were reviewed. Second, our study inclusion criteria meant that articles were removed from our analysis if they did not specify certain things, such as stroke subtypes. Third, this study summarized published results and did not attempt new analyses of the results nor the raw data underlying those results. Nonetheless, this systematic review used one of the most rigorous protocols for systematic reviews (i.e., PRISMA Guidelines) to assess traditional or conventional stroke risk factors extend to older individuals. Alternatively, Quality of Reporting of Meta-analyses (QUOROM) guidelines were not used because most of the studies were not Randomized Controlled Trials (RCT).

This systematic review provides evidence both for and against FSRP risk components for older adult populations, while also identifying other potentially important factors not included in the FSRP. It appears that risk factors (e.g., levels of serum androgens; C-reactive protein; advanced glycation endproducts; thrombin generation and increased left ventricular mass; hypertension; diabetes; physical activity) identified in the FSRP for younger-aged cohorts may be generalizable to older cohorts. Furthermore, this systematic review provides preliminary evidence that other risk factors not included in the FSRP may be salient for older populations. In addition, research needs to go a step further and examine even the oldest old, those who are 85+, as the projected average age of death continues to increase to almost 90 years by 2030 [46]. Lastly, the predictive factors that were identified for ischemic stroke may provide information for clinicians to utilize in assessing ischemic stroke risk, as well as creating protocols to address these risk factors.

## METHODS

We systematically reviewed risk factors for first ischemic stroke in older individuals, using the PRISMA guidelines. To focus on older adults, we included published studies that included participants age 65 years and older at baseline.

### Search strategy

A comprehensive search of PubMed, EMBASE and PsycINFO data bases was conducted by two researchers, to include articles published between the date of origin of each database through December 23, 2018. The search terms used were [“stroke” or “ischemic stroke” or “cerebrovascular accident” or “brain stem infarction” or “infarction, anterior cerebral artery” or “infarction, middle cerebral artery” or “infarction, basilar artery” or “infarction, brainstem” or “brain volume” or “thalamic stroke” or “putaminal stroke” or “cerebrovascular disorders” or “cerebral infarction” or “cerebellar infarction”] and [“risk factors” or “hypertension” or “diabetes mellitus” or “smoking” or “atrial fibrillation” or “dyslipidemia” or “previous myocardial infarction/ coronary artery disease” or “family history of myocardial infarction/ coronary artery disease” or “previous stroke/transient ischemic attack” or “family history of stroke/ transient ischemic attack” or “illicit drug use” or “alcohol” or “obesity” or “sleep apnea”] and [“Aged, 70 and Over” or “aging population” or “elderly” or “geriatric”]. Boolean logic was used to help maximize the number of relevant studies. We applied search parameters to limit the results to full-text. We applied search parameters to limit the results to full-text.

### Eligibility criteria

Selected for inclusion in this systematic review were: studies involving human subjects and case-control or prospective studies. These studies had to include ischemic stroke as an outcome, risk factor measurements at age >65 years, and had to be comprised of a study sample with mean age > 70 years. In addition, all reports had to be published in English. Degree of risk or protection associated with any given factor had to be presented using an estimate of Relative Risk (RR) an Odds Ratio (OR). Studies were excluded if they lacked primary data (e.g., editorials, review articles, or protocol papers), were not published in a peer-reviewed journal, were prevalence studies, focused on hemorrhagic stroke or transient ischemic attacks (TIA), examined risk factors for recurrent stroke, or did not specify the type of stroke outcome. Assessment of publication eligibility was performed independently in a blinded (to each other) standardized manner by two reviewers (JS; JM). A third reviewer (CC) resolved any disagreements.

### Quality assessment

We used the Newcastle-Ottawa quality assessment scale [10] for assessing the quality of studies in meta-analyses. The Newcastle-Ottawa scale rates the quality of categories pertaining to observational prospective longitudinal studies. Categories include participant selection, comparability, and outcome. Two reviewers independently assessed each study using the assessment scale (see Table 1). Any disagreements were resolved by consensus with the fifth author (JM). All authors had 100% agreement with the final results reported in Table 2. Inter-class correlation coefficients (ICC) were calculated to measure inter-rater reliability among reviewers using SPSS (IBM SPSS, Version 20, IBM Corp.)

## Supplementary Material

Appendix A. Newcastle-Ottawa Quality Assessment Scale
