# Supplementary material for: Independent ischemic stroke risk factors in older Americans: a systematic review
Source: Aging (Albany NY). 2019 May 24;11(10):3392–407. doi: 10.18632/aging.101987 (PMC6555455; doi:10.18632/aging.101987)
Supplement: Appendix A. Newcastle-Ottawa Quality Assessment Scale [file aging-11-101987-s001.pdf]

Appendix A. Newcastle-Ottawa Quality Assessment Scale.

	Selection				Comparability		Outcome	
	Representatives of Exposed Cohort	Selection of Nonexposed Cohort	Ascertainment of Exposure	Outcome Not Present	Comparability of Cohorts	Assessment of Outcome	Follow-up A	Follow-up B
**Serologic/Diagnostic**								
Kizer et al. (2014)	A	N/A	A	B	A	B	A	B
Cao et al., (2003)	A	N/A	A	B	A	B	A	B
Sacco et al., (2004)	B	N/A	A	A	A	B	A	B
Abbott et al., (2007)	B	N/A	A	A	A	B	A	B
Shores et al. (2014)	B	N/A	A	A	A	B	A	B
Khawaja et al., (2014)	A	N/A	A	B	A	B	A	B
Raipathak et al., (2011)	B	N/A	A	A	A	B	A	B
Saber et al., (2015)	A	N/A	A	A	A	B	A	B
Gardin et al., (2001)	A	N/A	A	B	A	B	A	B
**Traditional**								
Mukamel et al. (2005)	A	N/A	B	B	A	B	A	B
Sacco et al. (1999a)	A	N/A	B	B	A	C	N/A	N/A
Ottenbacher et al. (2004)	C	N/A	A	B	A	B	A	B
Naderi, Masoomi, Mozaffar and Malik (2014)	A	N/A	A	A	A	B	A	B
Lichtman, Krumbolz, Wang, Radford and Brass (2002)	A	N/A	A	B	A	B	A	B
Abbott et al. (2001)	A	N/A	A	B	A	B	A	B
Seshardi et al., (2001)	A	N/A	A	B	A	B	A	B
Colantonio, Kasl & Ostfield (1992)	A	N/A	B	B	A	B	A	B

Sacco et al. (1998)	A	N/A	A	A	A	B	N/A	N/A
Mukamel et al. (2005)	A	N/A	B	N/A	A	C	A	B
Genetic								
Ferrucci et al. (1997)	B	N/A	A	N/A	A	B	A	B
Brophy et al. (2006)	B	N/A	A	N/A	A	A	A	B
Olsen et al. (2015)	A	N/A	A	N/A	A	A	A	B
Psychosocial								
Arbelaez, Ariyo, Crum, Fried and Ford (2007)	A	N/A	B	B	A	B	A	B
Henderson et al., (2013)	A	N/A	B	B	A	B	A	B
Yu et al., (2015)	A	N/A	A	A	A	A	A	A
Cognitive/ Miscellaneous								
Ostir et al., (2003)	B	N/A	B	A	A	C	A	B
Elkins et al., (2004)	A	N/A	B	A	A	C	A	B
Ferruci et al., (1999)	A	N/A	B	A	A	C	A	B
Luchsinger et al., (2001)*	A	N/A	A	N/A	A	B	A	B

Selection:
1) Representativeness of the exposed cohort—A = truly representative of the average number of patients in the community; B = somewhat representative of the average number of patients in the community; C = selected group of users, e.g., nurses, volunteers; D = no description of the derivation of the cohort.
2) Selection of the nonexposed cohort—A = drawn from the same community as the exposed cohort; B = drawn from a different source; C = no description of the derivation of the nonexposed cohort.
3) Ascertainment of exposure—A = secure record (e.g., surgical records); B = structured interview; C = written self-report; D = no description.
4) Demonstration that outcome of interest was not present at start of study—A = yes; B = no.
Comparability:
1) Comparability of cohorts on the basis of the design or analysis—A = study controls for known risk factors of ischemic stroke (e.g., Age; Gender); B = study controls for any additional factor (these criteria could be modified to indicate specific control for a second important factor).
Outcome:
1) Assessment of outcome—A = independent blind assessment; B = record linkage; C = self-report; D = no description.
2) Was follow-up long enough for outcomes to occur—A = yes (select an adequate follow-up period for outcome of interest); B = no.
3) Adequacy of follow-up of cohorts—A = complete follow up, all subjects accounted for; B = small number of subjects lost to follow-up unlikely to introduce bias; C = no description of subjects lost; D = no statement on loss to follow-up.
